# Spontaneous cervical hematoma caused by giant parathyroid adenoma in an elderly patient: A case report

**DOI:** 10.3892/mi.2023.125

**Published:** 2023-11-30

**Authors:** Belén Matías-García, Fernando Mendoza-Moreno, Manuel Díez-Alonso, Enrique Ovejero-Merino, Cristina Vera-Mansilla, Alma Blázquez-Martín, Ana Quiroga-Valcárcel, Rubén Jiménez-Martín, Rebeca D'Amico, Inmaculada Lasa-Unzúe, Alberto Gutiérrez-Calvo

**Affiliations:** Department of General and Digestive Surgery, Príncipe de Asturias Teaching Hospital, 28805 Madrid, Spain

**Keywords:** parathyroid adenoma, thyroidectomy, cervical hematoma, primary hyperparathyroidism

## Abstract

Spontaneous cervical hematoma usually occurs as a consequence of extracapsular bleeding from a parathyroid gland, generally due to the presence of an adenoma (giant adenoma), glandular hyperplasia, cystic component, or, less frequently, due to the existence of a carcinoma. The hematoma can be confined to the cervical compartment or extend to the mediastinum, potentially causing airway compression. Despite this, the recommended management in hemodynamically stable patients consists of surveillance and hospital monitoring with delayed surgery after a few weeks. On the other hand, in those patients with airway compromise and instability, emergency surgery, consisting of cervicotomy and drainage, is mandatory. The present study describes the case of a 78-year-old patient with a medical history of high blood pressure, non-insulin-dependent diabetes mellitus, dyslipidemia, moderate aortic stenosis, chronic kidney disease and sarcoidosis under pharmacological treatment who attended the emergency department due to symptoms of neck pain, an increase in soft tissue, and dyspnea on moderate exertion with an evolution leading to respiratory failure. This was secondary to a diagnosis of spontaneous cervical hematoma that required urgent surgical intervention. The results of histopathological analysis revealed that a giant parathyroid adenoma was responsible for the bleeding. The patient had a complicated post-operative period with a prolonged admission to the intensive care unit.

## Introduction

Primary hyperparathyroidism (PHPT) is the most common pathological condition affecting the parathyroid glands and represents the third most common cause of endocrine disorders worldwide (1,2). It is estimated that the incidence of PHPT is ~2-4 cases per 10,000 inhabitants, with a higher prevalence among post-menopausal women (1). In 80-90% of cases, PHPT is attributed to the presence of a parathyroid adenoma, which autonomously produces parathyroid hormone (PTH), affecting calcium and phosphorus metabolism (3,4).

Spontaneous parathyroid hemorrhage is an exceptionally rare, yet potentially life-threatening condition due to airway compromise, often necessitating urgent surgical intervention. Clinically, it manifests with symptoms, such as cervical pain, dysphagia, dysphonia, or dyspnea, and physical examination may reveal ecchymosis in the cervical and/or thoracic regions (5).

The present study describes the case of a patient with spontaneous hemorrhage from a giant parathyroid adenoma resulting in acute airway compromise requiring surgical drainage.

## Case report

A 78-year-old female patient with a medical history of high blood pressure, non-insulin-dependent diabetes mellitus, dyslipidemia, moderate aortic stenosis, chronic kidney disease and sarcoidosis, receiving pharmacological treatment, presented to the Emergency Department of Príncipe de Asturias Teaching Hospital (Madrid, Spain) with complaints of increased cervical swelling, dyspnea during moderate exertion and odynophagia.

Her vital signs upon admission were as follows: Blood pressure, 185/88 mmHg; heart rate, 86 bpm; and baseline oxygen saturation, 96%. A physical examination revealed a cervical soft tissue hematoma without crepitation or fluctuation upon palpation. A cervical ultrasound was performed as part of the imaging assessment, which revealed a markedly enlarged thyroid with multiple nodules and intrathoracic extension, as well as increased subcutaneous tissue thickness with altered echogenicity, without distinct collections. A posteroanterior chest X-ray displayed a reduction in tracheal caliber in the cervical region related to a mixed multinodular goiter.

**Figure 1:**
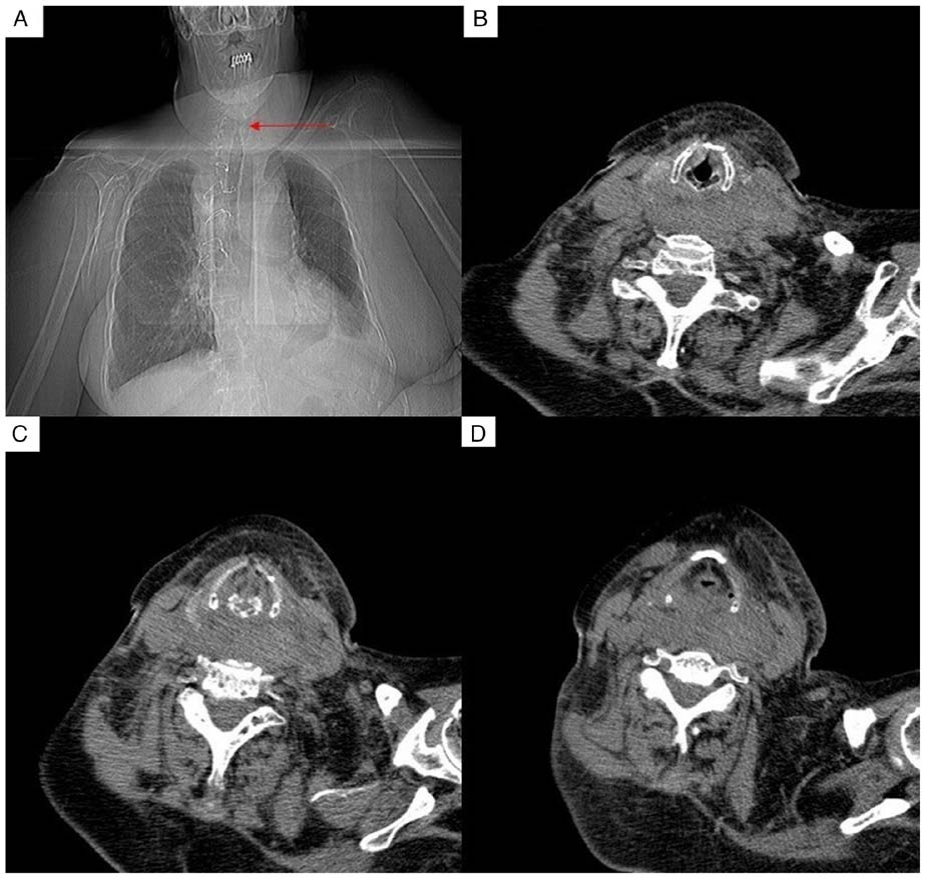
(A) Chest X-ray illustrating the tapering of the trachea (red arrow). (B-D) Cervical hematoma with progressive airway compression.

Due to the clinical suspicion of spontaneous cervical hematoma and the diagnosis of a hypertensive crisis, the patient remained under hospital observation. With stable clinical condition, blood pressure was managed through intravenous urapidil infusion and arterial phase control. However, during her stay in the emergency room, the patient experienced a desaturation episode with sudden respiratory distress (oxygen saturation decreased to 90%) and dysphonia. In response to this, a cervico-thoracic axial computed tomography (CT) scan with intravenous contrast was performed, revealing a lesion of heterogeneous density ~2x5 cm (anteroposterior x transverse) in the left thyroid lobe, extending to the second thoracic vertebra, with hyperdensity in different phases, suggestive of a hemorrhagic complication. Additionally, an increase in soft tissue density in the parapharyngeal and retropharyngeal space was observed, without signs of active bleeding (Fig. 1).

**Figure 2:**
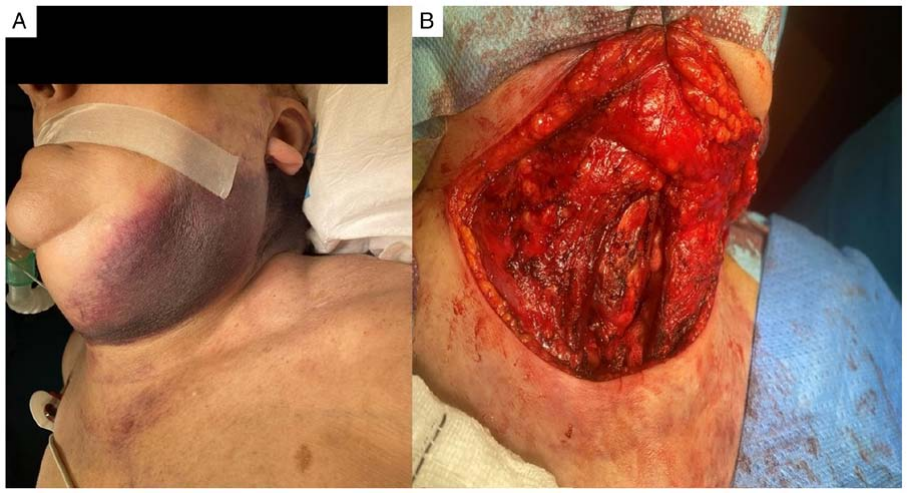
(A) Cervical spontaneous hematoma. (B) Cervicotomy surgical field following thyroidectomy and drainage of the cervical hematoma.

Given the worsening clinical condition of the patient and the potential for airway compromise, urgent surgery was deemed necessary. Under general anesthesia, following orotracheal intubation with a video laryngoscope, a median cervicotomy was performed. This revealed a large cervical hematoma extending into the mediastinum and parapharyngeal spaces, associated with the thyroid gland, leading to destruction and bleeding in the left thyroid lobe. The hematoma within the thyroid gland was evacuated, and a total thyroidectomy and tracheostomy were performed due to persistent bleeding (Fig. 2).

Following the surgery, the patient was admitted to the intensive care unit, where she experienced a complicated post-operative course marked by the development of pneumonia associated with mechanical ventilation and disruptions in phosphorus-calcium metabolism, including hypocalcemia refractory to replacement therapy and hypothyroidism. Following a 97-day hospital stay, the patient was discharged.

The histopathological examination of the surgical specimen revealed a thyroidectomy specimen weighing 15.8 g with dimensions of 4x4x12.5 cm, including a papillary microcarcinoma of the thyroid (1.5 mm) with clear surgical margins. Furthermore, the specimen contained parathyroid tissue measuring 6x4x3 cm and weighing 20.51 g, consistent with an adenoma and showing the presence of a hemorrhagic focus.

## Discussion

The parathyroid glands are small endocrine glands nestled in the neck, typically located behind the larger thyroid gland. While the majority of individuals have four parathyroid glands, the exact number can vary among individuals.

These glands play a pivotal role in regulating calcium levels in both the bloodstream and cells. Their primary function is to produce and secrete PTH, which plays a crucial role in maintaining the calcium and phosphorus levels in the body. They typically measure between 3-5 mm in size and have a weight of ~70 mg (6).

A parathyroid adenoma is a benign (non-cancerous) tumor that can affect one of the parathyroid glands. By contrast, a ‘giant’ parathyroid adenoma refers to a significantly enlarged parathyroid adenoma. While the majority of parathyroid adenomas are relatively small, with an average weight of <1 g, giant adenomas can be substantially larger, often weighing >3.5 g and measuring >2 cm in length. The largest reported giant parathyroid adenoma, as described in the study by Mahmodlou *et al* (1), measured 9x6x4 cm and weighed 122 g.

Giant parathyroid adenomas are a less common subtype of parathyroid adenomas, although they are still benign (non-cancerous) tumors. These adenomas overproduce PTH, leading to primary hyperparathyroidism, and they represent 5% of all parathyroid adenomas (7).

Spontaneous cervical hematoma is a rare complication of PHPT. The first documented case of spontaneous cervical hematoma due to a parathyroid adenoma dates back to 1934 when Capps (8) described it in the literature. Since then, <60 cases have been reported worldwide (3).

In the absence of cervical trauma, it is hypothesized that an imbalance between the gland's tissue growth and its blood flow may lead to phenomena, such as hemorrhage, infarction and subsequent necrosis, similar to the apoplexy observed in other endocrine neoplasms (9). Unlike the thyroid gland, the capsule surrounding the parathyroid glands is exceptionally thin, which can result in a rapid extension into the cervical and/or mediastinal regions in case of rupture (10,11).

Imaging tests, including CT scans, ultrasonography, or magnetic resonance imaging, are indispensable for diagnosing and pinpointing the location of the hematoma. The differential diagnosis should consider other conditions, such as cervical abscess, superior vena cava syndrome, cervical dissection, or subacute thyroiditis (5,12).

As regards the symptoms, patients with spontaneous cervical hematoma may experience neck swelling, tracheal and esophageal compression, and develop dysphagia, dyspnea and hoarseness. Simcic *et al* (13) proposed a more specific triad of symptoms, namely neck swelling, neck ecchymosis and hypercalcemia. However, elevated calcium levels have been observed in <70% of spontaneous hematoma cases. In the patient described herein, despite her chronic renal failure, neither hypercalcemia nor elevated PTH levels were found.

According to the study by Alegre *et al* (14), the management of patients with spontaneous cervical hematoma should be conservative if there is no airway compromise and hemodynamic instability persists. In such cases, intervention is typically delayed, involving procedures, such as thyroidectomy or parathyroidectomy, typically performed within ~6 weeks. This conservative approach has a success rate of 51.4% and is particularly suitable for elderly patients with high morbidity (3). In the case described in the present study, given the absence of hemodynamic instability, respiratory compromise and the notable morbidity of the patient, a non-operative approach involving monitoring and blood pressure control was initially favored. However, as the respiratory condition of the patient deteriorated, surgical intervention became necessary.

In conclusion, in the face of adversity, spontaneous cervical hematoma emerges as a rare yet formidable challenge, often originating from a distressed parathyroid gland (be it an adenoma, hyperplasia, cyst, or parathyroid carcinoma). It serves as a poignant reminder that within life's unpredictable narrative, the unexpected can occur.

The occurrence of a non-traumatic cervical hemorrhage, poses as a reminder of the resilience that resides within humans. The path to healing may take various forms, with options ranging from a prudent, watchful eye to timely surgical intervention. In this journey, the path chosen is a reflection of the patient's clinical stability, underscoring the importance of individualized care.

Emphasizing the importance of reminding clinical staff to be vigilant in recognizing cervical hematoma induced by parathyroid adenoma as a life-threatening condition cannot be overstated. While it is a rare occurrence, the consequences of overlooking this condition can be severe. Cervical hematoma in the context of parathyroid adenoma can lead to airway obstruction and respiratory compromise, rendering it an urgent medical emergency. This life-threatening situation underscores the critical need for early detection and immediate intervention. By raising awareness among clinical staff, healthcare professionals can ensure that timely assessments and treatments are administered, potentially saving lives in situations where every moment counts. Moreover, such reminders can help prevent misdiagnosis or delayed intervention, reducing the risk of adverse outcomes for patients with this uncommon, yet ‘high-stakes’ medical condition*.*

## Data Availability

The datasets used and/or analyzed during the current study are available from the corresponding author on reasonable request.
